# Skin manifestations after immunisation with an adjuvanted recombinant zoster vaccine, Germany, 2020

**DOI:** 10.2807/1560-7917.ES.2023.28.50.2300261

**Published:** 2023-12-14

**Authors:** Stefano Orru', Sibylle Bierbaum, Alexander Enk, Hartmut Hengel, Marcus Hoffelner, Daniela Huzly, Brigitte Keller-Stanislawski, Vera Mahler, Maja Mockenhaupt, Doris Oberle

**Affiliations:** 1 Division of Safety of Biomedicines and Diagnostics, Paul-Ehrlich-Institut, Langen, Germany; 2 Faculty of Medicine, University of Freiburg, Freiburg, Germany; 3 Department of Microbiology, Virology and Hygiene, Institute of Virology, Medical Center, University of Freiburg, Freiburg, Germany; 4 German Consulting Laboratory for HSV and VZV, Medical Center, University of Freiburg, Freiburg, Germany; 5 Department of Dermatology, University Hospital Heidelberg, Heidelberg, Germany; 6 Division of Allergology, Paul-Ehrlich-Institut, Langen, Germany; 7 Dokumentationszentrum schwerer Hautreaktionen (dZh), Department of Dermatology, Medical Center, University of Freiburg, Freiburg, Germany

**Keywords:** Shingrix, recombinant zoster vaccine, varicella-zoster virus, shingles, herpes zoster, skin manifestation

## Abstract

**Background:**

Shortly after the launch of a novel adjuvanted recombinant zoster vaccine (RZV), Shingrix, cases of suspected herpes zoster (HZ) or zoster-like skin reactions following immunisation were reported.

**Aim:**

We aimed to investigate if these skin manifestations after administration of RZV could be HZ.

**Methods:**

Between April and October 2020, general practitioners (GP) reporting a suspected case of HZ or zoster-like skin manifestation after RZV vaccination to the Paul-Ehrlich-Institut, the German national competent authority, were invited to participate in the study. The GP took a sample of the skin manifestation, photographed it and collected patient information on RZV vaccination and the suspected adverse event. We analysed all samples by PCR for varicella-zoster virus (VZV) and herpes-simplex virus (HSV) and genotyped VZV-positive samples. In addition, cases were independently assessed by two dermatologists.

**Results:**

Eighty eligible cases were enrolled and 72 could be included in the analysis. Of the 72 cases, 45 were female, 33 were 60–69 years old, 32 had skin symptoms in the thoracic and 27 in the cervical dermatomes. Twenty-seven samples tested PCR positive for VZV (all genotyped as wild-type, WT), three for HSV-1 and five for HSV-2.

**Conclusion:**

It may be difficult to distinguish HZ, without a PCR result, from other zoster-like manifestations. In this study, VZV-PCR positive dermatomal eruptions occurring in the first weeks after immunisation with RZV were due to WT VZV, which is not unexpected as HZ is a common disease against which the vaccine is unlikely to provide full protection at this time.

**Key public health message**

**What did you want to address in this study and why?**

Shingles, also known as herpes zoster (HZ), is a common disease and caused by the varicella-zoster virus (VZV). Affected patients have a painful skin rash with blisters. HZ can be prevented by vaccination. We wanted to study if zoster-like skin manifestations appearing closely after vaccination with the novel zoster vaccine Shingrix can be shingles.

**What have we learnt from this study?**

The majority of patients with skin manifestations after HZ vaccination in our study tested negative for VZV by polymerase chain reaction (PCR), some were positive for herpes-simplex virus type 1 or 2. Most skin lesions in patients with VZV detected occurred in the first weeks after the first dose of Shingrix and immediately or shortly after the second. The virus in the patients was not a vaccine strain.

**What are the implications of your findings for public health?**

It is difficult to diagnose HZ without testing for VZV. The few PCR-confirmed HZ cases due to wild-type VZV were not unexpected as HZ is a common disease against which Shingrix commits full protection only in case of completed immunisation schedule. Current vaccination recommendations should continue to be followed. No further risk minimisation measures are warranted.

## Introduction

Varicella-zoster virus (VZV), of the species of *Varicellovirus humanalpha* *3*, belongs to the subfamily of *Alphaherpesvirinae* and typically causes chickenpox and shingles, but also postherpetic neuralgia (PHN) [1,2].

After first infecting the epithelial cells of the individual, VZV is able to reside lifelong until reactivation, in various parts of the human body such as trigeminal and dorsal root ganglia and to even disseminate in visceral organs in severe cases [3]. Thus, individuals with a primary VZV infection (varicella) will acquire immunity against chickenpox but can still develop shingles (herpes zoster, HZ) if viral innate immune evasion and subversion strategies are activated, especially in case of waning VZV immunity. Due to viral spreading inside sensory neurons, VZV can reach the body surface again or the central nervous system, causing various symptoms and clinical manifestations [4].

In contrast to varicella, HZ occurs more frequently in older than in younger individuals and its incidence is higher in females compared with males [5-7].

For the prevention of HZ and PHN, the German Standing Committee on Vaccination (STIKO) recommends two 0.5 mL intramuscular injections of an adjuvanted recombinant zoster vaccine (RZV) (Shingrix, GlaxoSmithKline Biologicals S.A., Rixensart, Belgium), at an interval of at least 2 months and a maximum of 6 months [8]. This recommendation applies to individuals aged ≥ 60 years as a standard vaccination and to patients aged ≥ 50 years with specific underlying diseases or immunodeficiency as an indication-based vaccination [8-11].

A live zoster vaccine (ZVL) (Zostavax, Merck Sharp and Dohme B.V., BN Haarlem, the Netherlands) is not recommended by the STIKO as a standard vaccine due to its limited effectiveness and restricted duration of effect [12]. In addition, administration to immunocompromised or immunodeficient patients may result in disseminated disease, including fatal outcome [13].

The Paul-Ehrlich-Institut (PEI), as national competent authority responsible for the safety of vaccines and biomedicines in Germany, receives reports of adverse events following immunisation (AEFI) with vaccines via the public health authorities in accordance with the Protection Against Infection Act (Infektionsschutzgesetz, IfSG) [14]. Physicians are legally obliged to report AEFIs, i.e. health complaints that go beyond the usual vaccination reactions and are not evidently due to other causes, to the competent public health department, which in turn reports them immediately and in pseudonymised form (i.e. without providing the patient’s name and address) to the PEI. In addition, the PEI receives AEFI reports from the Drug Commission of German Pharmacists (Arzneimittelkommission der Deutschen Apotheker, AMK) and the Drug Commission of the German Medical Association (Arzneimittelkommission der deutschen Ärzteschaft, AkdÄ), since pharmacists and physicians have a professional obligation to report AEFIs, respectively. According to the German Medicinal Products Act (Arzneimittelgesetz, AMG), marketing authorisation holders have an obligation to report via the European adverse drug reaction database EudraVigilance [15]. The German reports go from there to the PEI.

In addition, healthcare professionals and vaccinated persons or their relatives can report AEFIs directly to the PEI. Reports can be made by post, email, telephone or online via the reporting portal of the PEI (www.nebenwirkungen.bund.de). At the PEI, identical reports from different sources are merged into one case.

In compliance with the AMG, the PEI is obliged to report AEFIs electronically at certain intervals in an internationally standardised format and pseudonymised to the joint EudraVigilance database of the European Medicines Agency, to which every regulatory authority in the EU has access.

The reporting system for AEFIs after vaccination against HZ and any other diseases is similar.

Shingrix was launched on the German market on 1 May 2018 [8]. Between January 2019 and December 2019, 53 suspected cases of HZ or zoster-like, in parts vesicular, skin eruptions that occurred shortly after RZV administration were reported to the AkdÄ [16].

In April 2020, the PEI initiated a multicentre study to investigate if suspected cases of HZ and zoster-like skin manifestations in close temporal association with the administration of RZV could be HZ.

## Methods

### Case reporting

The PEI in close collaboration with the AkdÄ informed about the study on their web pages and by circulating a Drug Safety Mail to all physicians in Germany [17].

General practitioners (GP) had the possibility to promptly notify suspected RZV-associated AEFIs either by phone or by fax or email. Subsequently, they were asked to fill in and return the standard adverse event form (AEF) [18] in accordance with section 6 subsection 1 number 3 of the IfSG [14]. The notification of cases was identical with the standard reporting of AEFIs.

All completed AEFs received were pseudonymised and stored in the PEI database for adverse events (VigilanceOne, 2003–2015 PharmApp Solutions GmbH, http://www.PharmApp.de, Germany, version 2.4.11.1).

### Case recruitment

Recruitment of study cases started on 15 April 2020 and ended on 14 October 2020.

All potential study cases notified within this time frame underwent a screening process according to the eligibility requirements described in the study protocol.

The inclusion criteria were:

(i) suspected case of HZ or zoster-like skin manifestation,

(ii) temporal relationship between symptom onset and RZV vaccination, and

(iii) sufficiently informative AEF completed by a physician.

The exclusion criteria were:

(i) no sample from the skin manifestation,

(ii) time to symptom onset > 28 days after immunisation with RZV, and

(iii) AEFI reported by a private person.

The GP was contacted and asked in a timely manner to participate in the study, send a written confirmation of the agreement to participate and to obtain the patient’s written consent. Potential cases that did not meet the inclusion criteria or had exclusion criteria were not considered.

The PEI provided all participating GPs with a patient informed consent form (ICF), a study registration form, a case report form (CRF) and a kit for the collection (including instructions for use) and transport of the patient specimen via a courier (GO! Express and Logistics Deutschland GmbH, Bonn, Germany).

The following steps were done only for those cases with an ICF signed by the patient and a registration form signed by the GP.

### Case report form

The paper CRF, filled by the GP, contained 14 questions concerning the patient: age group, sex, first digit of the postcode, medical history, immune status, concomitant medication or vaccination, vaccination date, dose number, route of administration, injection site and batch number of the RZV, start and end date, type, localisation, clinical course and treatment of the suspected AEFI and the laboratory diagnostics performed, if available.

### Sample collection and processing

The GPs were not provided with any specific training for assessing skin eruptions, but they received written and illustrated instructions for correct specimen collection [19] and transport to the German Consulting Laboratory for Herpes-Simplex Virus (HSV) and VZV for analysis. A smear was taken by the GP by rolling a swab (eSwab, Copan Liquid Amies Elution Swab, Copan Italia S.p.A., Brescia, Italy) over the affected skin area according to the instructions [19]. After sampling, the swab was stored in the transport tube at room temperature and sent via a courier, in most cases within 24 hours, together with a sample submission form to the German Consulting Laboratory for HSV and VZV for analysis.

Samples of all enrolled cases were tested for the presence of VZV and HSV (type 1 and 2) by PCR with a limit of 73 copies/mL for VZV, 34 copies/mL for HSV type 1 and 73 copies/mL for HSV type 2 [20]. In previous studies, PCR-based approaches for detection of VZV DNA in clinical specimens obtained from vesicular eruptions have had sensitivities and specificities of almost 95% and 100%, respectively [21-23]. False-negative results caused by PCR inhibition could be avoided by including an internal amplification control [24]. Temperature conditions or storage time show relatively limited influence on the viral DNA stability in eSwab samples [25].

In case of VZV-positive results, PCR with primers for single nucleotide polymorphisms (SNP) located in open reading frames (ORF) 6 (SNP 5745) and 62 (SNP 105544, 105705, 107136 and 107252) of the VZV genome were used to differentiate the vaccine strain from wild-type (WT) VZV strains. The PCR products were sequenced and evaluated at the indicated variable positions [26,27]. In case of a VZV-negative result, day 14 was selected as the cut-off between PCR still sensitive (time window between symptom onset and sample collection ≤ 14 days) and not sensitive enough (time window between symptom onset and sample collection > 14 days).

The laboratory findings were promptly communicated to the GPs to give them the possibility to adjust the treatment accordingly, e.g. with an antiviral medication in case of a VZV-positive test result.

### Case validation

Two dermatologists (A.E. and M.M.) validated each case by assessing the AEF, the CRF, the laboratory findings and the digital photos of the skin manifestations, if available, blinded to the patient and the GP identity. The dermatologists confirmed or disproved the HZ diagnosis; in the latter case, they were asked to propose a differential diagnosis.

Cases with discordant diagnoses were validated by a third dermatologist (V.M.).

### Data management

Completed and returned AEFs, CRFs, laboratory results and dermatologists’ validations underwent an in-house review. Study data were pseudonymised and stored in an offline password-protected FileMaker Pro relational database (Claris International Inc., Santa Clara, United States (US), version 18.0.3). Only in-house data access was possible. No third parties had access to the database. In order to achieve good data quality, comprehensive computer-assisted plausibility checks were performed. In case of inconsistencies or missing information, the GPs, the laboratory staff or the dermatologists were contacted.

### Statistical analyses

Absolute and relative frequencies as well as median and interquartile range were calculated for qualitative and quantitative variables, respectively. To test for differences between VZV-positive and VZV-negative cases, chi-square tests or Fisher’s exact tests and Wilcoxon two sample tests were performed for qualitative and quantitative variables, respectively. A p value < 0.05 was considered statistically significant. Due to the explorative character of the study no *α* adjustment was made.

The statistical analysis was conducted using SAS (Statistical Analysis System, SAS Institute Inc., Cary, US, version 9.4).

## Results

### Suspected cases

**Figure 1:**
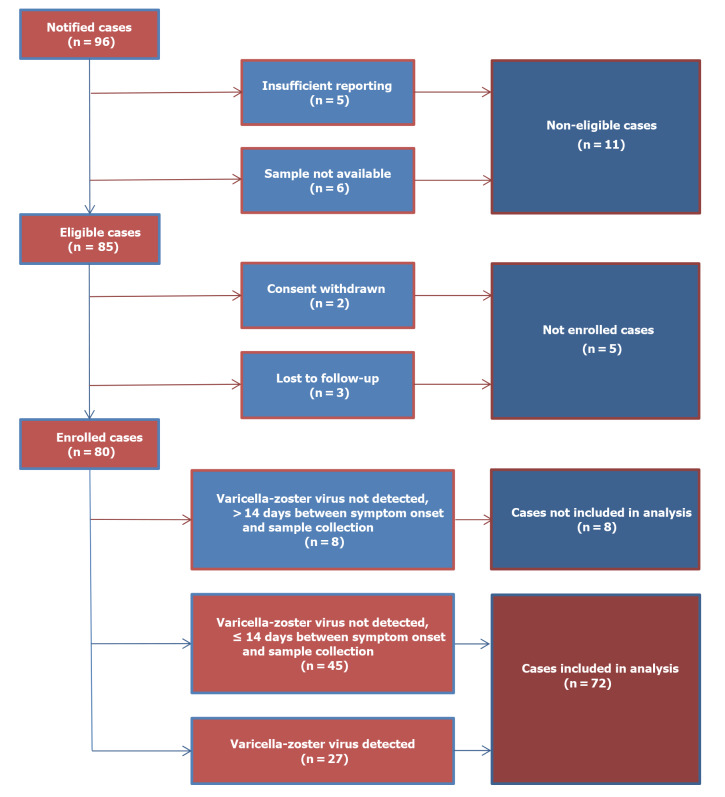
Flowchart of data processing in a study on skin manifestations after immunisation with an adjuvanted recombinant zoster vaccine, Germany, 2020 (n = 96)

Among all notifications of suspected cases of HZ or zoster-like skin manifestations after immunisation with RZV between April and October 2020, the PEI received 96 study participation requests (Figure 1).

Of these 96 suspected cases, 11 did not meet the eligibility criteria: five (5.2%) had an inconclusive AEF and six (6.3%) had no sample available for laboratory analysis. For two (2.1%) eligible suspected cases either the patient or the GP withdrew the consent and three (3.1%) were lost to follow-up.

After screening, 80 (83.3%) suspected cases reported by 78 GPs were enrolled. One GP included two different patients during the 6-month recruitment period, while another GP enrolled twice the same patient after experiencing AEFI after both the first and the second dose of the RZV.

Of these 80 enrolled suspected cases, 53 (66.3%) tested negative for VZV. However, eight of these cases were excluded from the analysis because the sample was not collected within 14 days after symptom onset. Of the 27 samples that were PCR positive for VZV, four were collected more than 14 days after the onset of symptoms. These cases were included in the study analysis because there was no reason to doubt the VZV-PCR positive results.

Finally, 72 (75.0%) suspected cases reported by 71 GPs were included in the analysis. The first case was enrolled on 27 April 2020 and the last one on 12 October 2020. Validation of the last case was completed on 16 November 2021.

### Study participants

More than half (45/72) of the study participants were female and most (42/72) were younger than 70 years (Table 1). The youngest patient was a 34-year-old woman with multiple sclerosis, vaccinated in line with the marketing authorisation.

**Table 1:** Demographic characteristics of cases with skin manifestations after immunisation with an adjuvanted recombinant zoster vaccine, Germany, 2020 (n = 72)

Characteristics of participants	VZV-positive cases^a^	VZV-negative cases^c^	Total		p value
	n	n	n	%	
Number of cases	27	45	72		NA
Sex					
Male	9	18	27	37.5	0.572
Female	18^b^	27	45^b^	62.5	
Age groups (years)					
≤ 49	0	1	1	1.4	NA
50–59	3	5	8	11.1	
60–69	10	23	33	45.8	
70–79	10^b^	13	23^b^	31.9	
80–89	4	3	7	9.7	
Combined age groups (years)					
≤ 69	13	29	42	58.3	0.175
70–89	14^b^	16	30^b^	41.7	
Geographic area^d^					
North	7	13	20	27.8	0.120
Centre	11	26	37	51.4	
South	9^b^	6	15^b^	20.8	

HSV: herpes-simplex virus; NA: not applicable; VZV: varicella-zoster virus.

^a^ All VZV-positive samples were genotyped to wild-type VZV strains.

^b^ One patient sample was PCR positive for VZV and HSV-1.

^c^ Two VZV-negative samples were PCR positive for HSV-1 and five for HSV-2.

^d^ Geographic area (first digit of the German postcode): north (1, 2 and 4), centre (0, 3, 5 and 6) and south (7, 8 and 9). A map is shown in Supplement (Supplementary Figure S1).

Percentages not included where n < 50.

Half (37/72) of the patients lived in central Germany. A map is shown in Supplement (Supplementary Figure S1).

Most analysed cases with information available, had no previous HZ episodes (47/69) or immune defects or deficiencies (56/68) in their medical history (Table 2). The minority (12/68) of participants reported a congenital or acquired immunodeficiency, immunosuppression or other underlying disease such as multiple sclerosis, psoriasis vulgaris, rheumatoid arthritis, essential thrombocythaemia, lymphoma, ovarian or prostate cancer, renal failure, ulcerative colitis, polymyalgia rheumatica or microscopic polyangiitis.

**Table 2:** Medical history, course of illness and outcome of cases with skin manifestations after immunisation with an adjuvanted recombinant zoster vaccine, Germany, 2020 (n = 72)

Characteristics of cases	VZV-positive cases^a^	VZV-negative cases^c^	Total		p value
	n	n	n	%	
Number of cases	27	45	72		NA
Previous herpes zoster episodes					
Number of responses	25	44	69		NA
At least one	5	17	22	31.9	0.110
1	4	9	13	18.8	NA
2–5	1	5	6	8.7	
6–10	0	1	1	1.4	
≥ 11	0	2	2	2.9	
None	20^b^	27	47^b^	68.1	
Immune defect or deficiency					
Number of responses	25	43	68		NA
Yes	4	8	12	17.6	1.000
Vaccine dose					
First	21^b^	29	50^b^	69.4	0.509
Second	6	16	22	30.6	
Time to symptoms onset (days)					
After any vaccine dose					
0–7	20	28	48	66.7	0.302
8–28	7^b^	17	24^b^	33.3	
After first vaccine dose					
Number of responses	21	29	50		NA
0–7	15	16	31	62.0	NA
8–14	3^b^	9	12^b^	24.0	
15–21	2	4	6	12.0	
22–28	1	0	1	2.0	
After second vaccine dose					
Number of responses	6	16	22		NA
0–7	5	12	17		NA
8–14	1	2	3		
15–21	0	0	0		
22–28	0	2	2		
Concomitant medication^d^					
Other vaccine	1	1	2	2.8	1.000^e^
Immune suppressant or modulator	2	5	7	9.7	0.704^e^
Nonsteroidal anti-inflammatory	6	11	17	23.6	0.830
Antiviral treatment					
Yes	11	8	19	26.4	**0.032**
Complication					
Number of responses	25	44	69		NA
Yes	6	4	10	14.5	0.152^e^
Hospitalisation					
Number of responses	26	44	70		NA
Yes	2	2	4	5.7	0.624^e^
Outcome (on day of last reporting)					
Number of responses	25	43	68		NA
Recovered or improved	16	42	58	85.3	< **0.001^e^**
Recovered	13^b^	38	51^b^	75.0	NA
Improved	3	4	7	10.3	
Not recovered	7	1	8	11.8	
Recovered with sequelae	2	0	2	2.9	

HSV: herpes-simplex virus; NA: not applicable; VZV: varicella-zoster virus.

^a^ All VZV-positive samples were genotyped to wild-type VZV strains.

^b^ One patient sample was PCR positive for VZV and HSV-1.

^c^ Two VZV-negative samples were PCR positive for HSV-1 and five for HSV-2.

^d^ Multiple answers possible.

^e^ Fisher’s exact test.

Entries in bold represent statistically significant findings.

Percentages not included where n < 50.

Two (2.8%) cases had received shortly after RZV concomitant vaccines against tick-born encephalitis (FSME-IMMUN, Pfizer Pharma GmbH, Berlin, Germany) or hepatitis A virus infection (Havrix, GlaxoSmithKline GmbH & Co. KG, München, Germany), seven (9.7%) had received immunosuppressive or immunomodulatory medication and 17 (23.6%) nonsteroidal anti-inflammatory drugs. No patient had been immunised with ZVL.

### Adverse events following immunisation

The most affected dermatomes were in the thoracic (32/72) and the cervical (27/72) area (Table 3). More than one dermatome was involved in nine (12.5%) cases. Localisation of the skin manifestation in the cervical area was statistically more frequently reported in VZV-negative cases (22/45) than in VZV-positive cases (5/27) (p = 0.010).

**Table 3:** Description of adverse events of cases with skin manifestations after immunisation with an adjuvanted recombinant zoster vaccine, Germany, 2020 (n = 72)

Characteristics of adverse events	VZV-positive cases^a^		VZV-negative cases^c^		Total		p value
	n		n		n	%	
Number of cases	27		45		72		NA
Dermatome location^d^							
Cervical	5		22		27	37.5	**0.010**
Thoracic	13		19		32	44.4	0.624
Lumbar	7^b^		5		12^b^	16.7	0.117^e^
Sacral	4		6		10	13.9	1.000^e^
Skin eruption^d^							
Erythema	17^b^		25		42^b^	58.3	0.537
Macules	0		9		9	12.5	**0.022** ^e^
Papules	7		15		22	30.6	0.509
Vesicles	21^b^		23		44^b^	61.1	**0.025**
Pustules	10^b^		13		23^b^	31.9	0.473
Haemorrhagic blisters	2		1		3	4.2	0.552^e^
Crusts	8		13		21	29.2	0.947
Other symptoms							
Number of responses	26		44		70		NA
Severe pain with skin eruption	20^b^		19		39^b^	55.7	**0.006**
Severe pain without skin eruption	4		3		7	10.0	0.411^e^
Neurological symptom	3		3		6	8.6	0.664^e^
Pruritus	16		34		50	71.4	0.159
Paraesthesia	17		17		34	48.6	**0.031**
Number of responses	25		44		69		NA
Other	8^b^		12		20^b^	29.0	0.677
Duration (days)	Median	IQR	Median	IQR	Median	IQR	p value
Skin eruption	19.0	15.0–31.0	11.0	8.0–15.0	13.0	9.0–20.0	**0.002**
Severe pain with skin eruption	20.0	12.0–35.0	7.0	4.0–16.0	12.0	7.0–22.0	**0.002**
Severe pain without skin eruption	37.0	NA	20.0	1.0–38.0	28.5	10.5–37.5	1.000
Neurological symptom	36.0	35.0–37.0	20.0	11.0–38.0	35.0	20.0–37.0	0.773
Pruritus	26.0	12.0–35.0	9.0	4.0–14.0	12.0	7.0–24.0	**0.006**
Paraesthesia	35.0	15.0–47.0	8.0	5.0–13.0	12.5	7.0–36.0	**0.001**
Other	19.0	9.0–37.0	3.5	1.5–9.5	9.0	2.0–17.0	**0.012**

HSV: herpes-simplex virus; IQR: interquartile range; VZV: varicella-zoster virus.

^a^ All VZV-positive samples were genotyped to wild-type VZV strains.

^b^ One patient sample was PCR positive for VZV and HSV-1.

^c^ Two VZV-negative samples were PCR positive for HSV-1 and five for HSV-2.

^d^ Multiple answers possible.

^e^ Fisher’s exact test.

Entries in bold represent statistically significant findings.

Percentages not included where n < 50.

The features of the skin eruptions were described as erythema, macules, papules, vesicles, pustules, haemorrhagic blisters and crusts. Vesicles were significantly more frequent among VZV-positive cases (21/27) than among VZV-negative cases (23/45) (p = 0.025), whereas macules were only reported in VZV-negative cases (9/45) (p = 0.022).

Other AEFIs reported included severe pain with or without skin eruption, neurological symptoms, pruritus and paraesthesia. Severe pain with skin eruption (20/27 VZV-positive cases vs 19/45 VZV-negative cases; p = 0.006) and paraesthesia (17/27 VZV-positive cases vs 17/45 VZV-negative cases; p = 0.031) were significantly more common in VZV-positive cases. Except for severe pain without skin eruption and neurological symptoms, the AEFIs persisted statistically longer in VZV-positive cases than in VZV-negative cases.

In 50 (69.4%) cases skin manifestations occurred after the first dose of RZV and in 22 (30.6%) cases after the second (Table 2).

In 48 (66.7%) of the 72 analysed cases, irrespective of dose number, symptom onset was within the first week and less often in the second, third or fourth week after immunisation.

**Figure 2:**
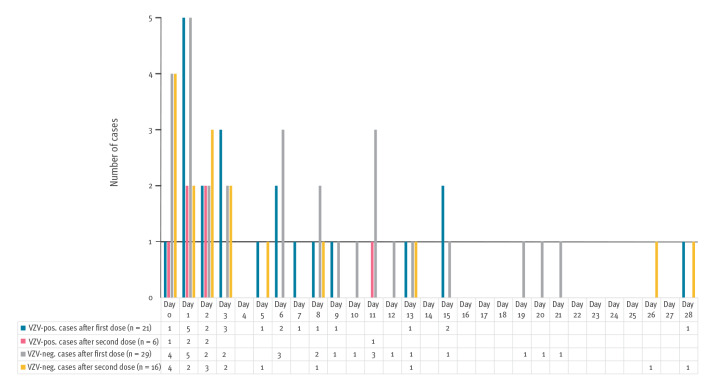
Time to symptom onset of cases with skin manifestations after immunisation with an adjuvanted recombinant zoster vaccine, Germany, 2020 (n = 72)

Vaccine dose or time to symptom onset did not vary between VZV-positive and VZV-negative cases (Figure 2).

neg.: negative; pos.: positive; VZV: varicella-zoster virus.

Ten (14.5%) of 69 patients experienced one or more of the following complications: PHN, HZ ophthalmicus, transient ischaemic attack, diplopia, exsiccation, arthralgia, myalgia, asthenia, headache and radiating pain (Table 2).

Four (5.7%) of 70 patients with available information were hospitalised and two (2.9%) of 68 patients reported sequelae (PHN with severe pain). According to section 4 subsection 13 of the AMG, those six AEFIs were defined as serious [15].

The outcome was significantly more often reported as ‘recovered or improved‘ for VZV-negative cases (42/43) than for VZV-positive cases (16/25) (p < 0.001).

### Molecular analyses

Twenty-one patients (including one case of coinfection with HSV-1) tested VZV-positive after the first dose of RZV and six after the second (Table 2). The virus of all positive cases was genotyped to WT, the DNA of the ZVL strain was not confirmed by sequencing analysis.

Herpes-simplex virus type 1 was detected in two of the 45 VZV-negative cases and HSV-2 in five.

Of the 19 analysed cases treated with antiviral drugs, virostatic agents such aciclovir or brivudin were given to more VZV-positive cases (11/27) than negative ones (8/45) (p = 0.032). However, HSV was detected only in two of the eight VZV-negative cases.

### Suspected diagnoses

Fifty-two (72.2%) of the 72 analysed cases were initially diagnosed by the participating GP as HZ or varicella but VZV was not detected by PCR in half of these cases (n = 26) (Table 4).

**Table 4:** Diagnoses of cases with skin manifestations after immunisation with an adjuvanted recombinant zoster vaccine by general practitioners and dermatologists, Germany, 2020 (n = 72)

Diagnoses of cases	VZV-positive cases^a^	VZV-negative cases^c^	Total	
	n	n	n	%
Number of cases	27	45	72	
By general practitioners				
HZ or varicella	26^b^	26	52^b^	72.2
No HZ or varicella	1	19	20	27.8
By two dermatologists				
HZ	25^b^	0	25^b^	34.7
HSV	0	9	9	12.5
Exanthema	0	2	2	2.8
Eczema	0	2	2	2.8
Folliculitis	0	1	1	1.4
Discordant diagnoses	2	2	4	5.5
Discordant differential diagnoses	0	29	29	40.3
By third dermatologist				
Number of responses	2	2	4	
HZ	1	0	1	
Non-assessable	1	2	3	

HSV: herpes-simplex virus; HZ: herpes zoster; VZV: varicella-zoster virus.

^a^ All VZV-positive samples were genotyped to wild-type VZV strains.

^b^ One patient sample was PCR positive for VZV and HSV-1.

^c^ Two VZV-negative samples were PCR positive for HSV-1 and five for HSV-2.

Percentages not included where n < 50.

Two dermatologists independently confirmed the classic form of HZ in 25 of the 27 VZV-positive cases. No case was diagnosed with HZ disseminatus or HZ sine herpete.

A third dermatologist confirmed the HZ diagnosis in one discordant case. The other discordant case was classified as non-assessable due to missing photo documentation although HZ disseminatus was suspected.

The most common concordant diagnosis of the 45 VZV-negative cases was HSV (n = 9), followed by exanthema (n = 2), eczema (n = 2) and folliculitis (n = 1). Discordant differential diagnoses were attributed to 29 of the 45 VZV-negative cases.

For two VZV-negative cases the dermatologists disputed the correctness of the PCR test result. The third dermatologist could not assess these cases due to missing information.

## Discussion

Herpes zoster is a significant health problem which impairs the quality of life of those affected, especially in the older generation and in individuals with immunodeficiency or being immunosuppressed. Effective primary prevention measures are needed.

The study was initiated in 2020 to examine a potential safety signal raised in 2019 in Germany when several suspected cases of HZ and zoster-like skin manifestations shortly after RZV administration were reported to the AkDÄ [16]. We tested swabs from the affected skin regions for VZV, HSV-1 and HSV-2 using PCR and dermatologically assessed the diagnoses made by the GPs.

When assessing the diagnoses, a heterogeneous picture was seen. In 37.5% of the 72 analysed cases VZV was detected by PCR. A WT VZV was identified in all VZV-positive samples, which is in line with the fact that no patient had previously been immunised with ZVL. In most (62.5%) cases, VZV was not detected. Fourteen of these 45 VZV-negative cases were concordantly diagnosed with HSV, exanthema, eczema or folliculitis by two independent dermatologists.

Thus, it is difficult to distinguish prima vista HZ from other zoster-like skin manifestations without PCR. In general practice, smears are rarely taken when HZ is suspected. Most often, the diagnosis is made based on the clinical presentation. However, in this study, half (26/52) of the cases initially assessed as HZ or varicella by GPs were not confirmed upon further examination. Therefore, caution should be taken when studies on HZ are conducted solely on the basis of secondary data, usually International Classification of Diseases (ICD) diagnosis codes (ICD-10) used by physicians and health insurers for billing purposes [28].

Twenty-one of the 27 cases tested VZV-positive after the first RZV dose, while the remaining six were detected within 14 days after the second dose, thus full vaccination. While the vaccine is ca. 90% effective in randomised clinical trials after the second dose of RZV, usually given at an interval of 2–6 months after the first dose, the effectiveness after the first dose is not known and is likely to be lower as measured by immunogenicity data [29]. These VZV-positive cases may therefore be concomitant VZV manifestations before developing a full protective vaccination response.

So far, HZ has only sporadically been reported after vaccination with RZV [30-33]. In two studies (ZOE-50 and ZOE-70) no increased incidence of HZ, herpes simplex or rash within 30 days after vaccination with RZV was observed compared with placebo (http://www.gsk-clinicalstudyregister.com; IDs 110390 and 113077). Well-designed and well-powered studies on this potential safety concern (including individual case verification) are still lacking.

Our study covered the entire country of Germany and suspected cases of HZ or zoster-like skin manifestations after RZV immunisation were reported from the whole country during a recruitment period of 6 months.

We conducted this case series according to a study protocol using objective diagnostic methods (i.e. PCR and genotyping) and a dermatological validation. All GPs received a standardised sampling kit (including instructions for use) and all samples were tested by the German Consulting Laboratory for HSV and VZV. Weather and transport conditions such as temperature and delivery time should not have affected the laboratory results [25].

Enrolled patients directly benefited from the laboratory findings which helped the GPs choose an appropriate treatment (use or discontinuation of antiviral therapy, in case of a VZV-PCR positive or negative result, respectively).

Our study was carried out during the COVID-19 pandemic which might have resulted in underreporting of AEFIs and undetected COVID-19 infections could have influenced the results. However, it should be noted that the variants of Severe Acute Respiratory Syndrome Coronavirus type 2 (SARS-CoV-2), which were often associated with a more severe disease pattern, were prevalent in 2020.

The exact number of RZV doses administered in Germany is not available. Between the first quarter of 2019 and the first quarter of 2021, the vaccination rate for HZ in persons aged ≥ 60 years was 5.0% for the first dose and 3.3% for the second [34]. During the recruitment period (April–October 2020), one suspected case of HZ was reported within 28 days of ZVL vaccination in a 68-year-old person. However, it is important to consider that ZVL is rarely used in Germany as RZV is recommended by the STIKO and the exact number of ZVL doses administered is unknown.

None of the enrolled participants was aged ≥ 90 years. In Germany, while the HZ incidence is 6.21 per 1,000 individuals among persons aged 50–59 years, the incidence is twice as high (13.19 per 1,000 individuals) in 90-year-olds [7].

The PCR test results could be false-negative in case of improper specimen collection, handling or transport. Also, we do not know how many skin eruptions were swabbed in patients with more than one skin eruption. Furthermore, PCR analyses were performed only for the detection of VZV and HSV. No testing was performed for other viruses or bacteria.

Symptom onset does not necessarily mean eruption onset. However, in most (79.2%) analysed cases, the symptom onset was the same as eruption onset, in 14 cases (eight of which were VZV-positive and in the remaining six VZV-negative cases the interval between eruption onset and sample collection was ≤ 8 days) the symptom onset was not the day of eruption onset and in one VZV-positive case the date of eruption onset was missing. In seven of the eight cases excluded from analysis, symptom onset coincided with eruption onset, but not in the remaining case (in which both the interval between symptom onset and sample collection and the interval between eruption onset and sample collection were > 14 days).

There was no indication of a difference in the clinical presentation of HZ after the introduction of RZV on the German market compared with before its introduction, but this was not the aim of this study (we did not have an unvaccinated control group). We cannot exclude the possibility that diagnosing classic forms of HZ in the GP setting only based on clinical presentation is more accurate than other forms of HZ, as the clinical picture may be more typical. This may have led to an information bias.

Case validation by the dermatologists was based on the information provided by the GPs. Comprehensive (complete) records were only partially available for some enrolled cases included in the analysis, which may have led to information bias. In addition, dermatologists could not examine the patients themselves. The above-mentioned points may possibly explain the few discordant dermatological diagnoses.

There may be superior study designs, such as population-based cohort studies based on large, linked databases. This study demonstrates that an individual case ascertainment using PCR analysis is indispensable.

## Conclusion

It may be difficult to distinguish HZ without a PCR result from other zoster-like manifestations. This study shows that VZV-PCR positive dermatome eruptions occurring in the first weeks after RZV dose 1 and immediately or shortly after dose 2 are due to WT VZV, which is not unexpected as this is a common disease against which the vaccine would not yet be expected to provide protection. Therefore, HZ may still occur after either RZV dose.

The benefits of RZV far outweigh the risks. German GPs should continue to follow the recommendations of the STIKO.

## Supplementary Data

620000 Supplementary Material
