# Supplementary material for: Skin manifestations after immunisation with an adjuvanted recombinant zoster vaccine, Germany, 2020
Source: Euro Surveill. 2023 Dec 14;28(50):2300261. doi: 10.2807/1560-7917.ES.2023.28.50.2300261 (PMC10831415; doi:10.2807/1560-7917.ES.2023.28.50.2300261)

SUPPLEMENTARY FIGURE S1. Geographic area according to first digit of the postcode, Germany, 2020 (n = 10)

North (1, 2 and 4), centre (0, 3, 5 and 6) and south (7, 8 and 9).

This supplementary material is hosted by Eurosurveillance as supporting information alongside the article Skin manifestations after immunisation with an adjuvanted recombinant zoster vaccine, Germany, 2020, on behalf of the authors, who remain responsible for the accuracy and appropriateness of the content. The same standards for ethics, copyright, attributions and permissions as for the article apply. Supplements are not edited by Eurosurveillance and the journal is not responsible for the maintenance of any links or email addresses provided therein.

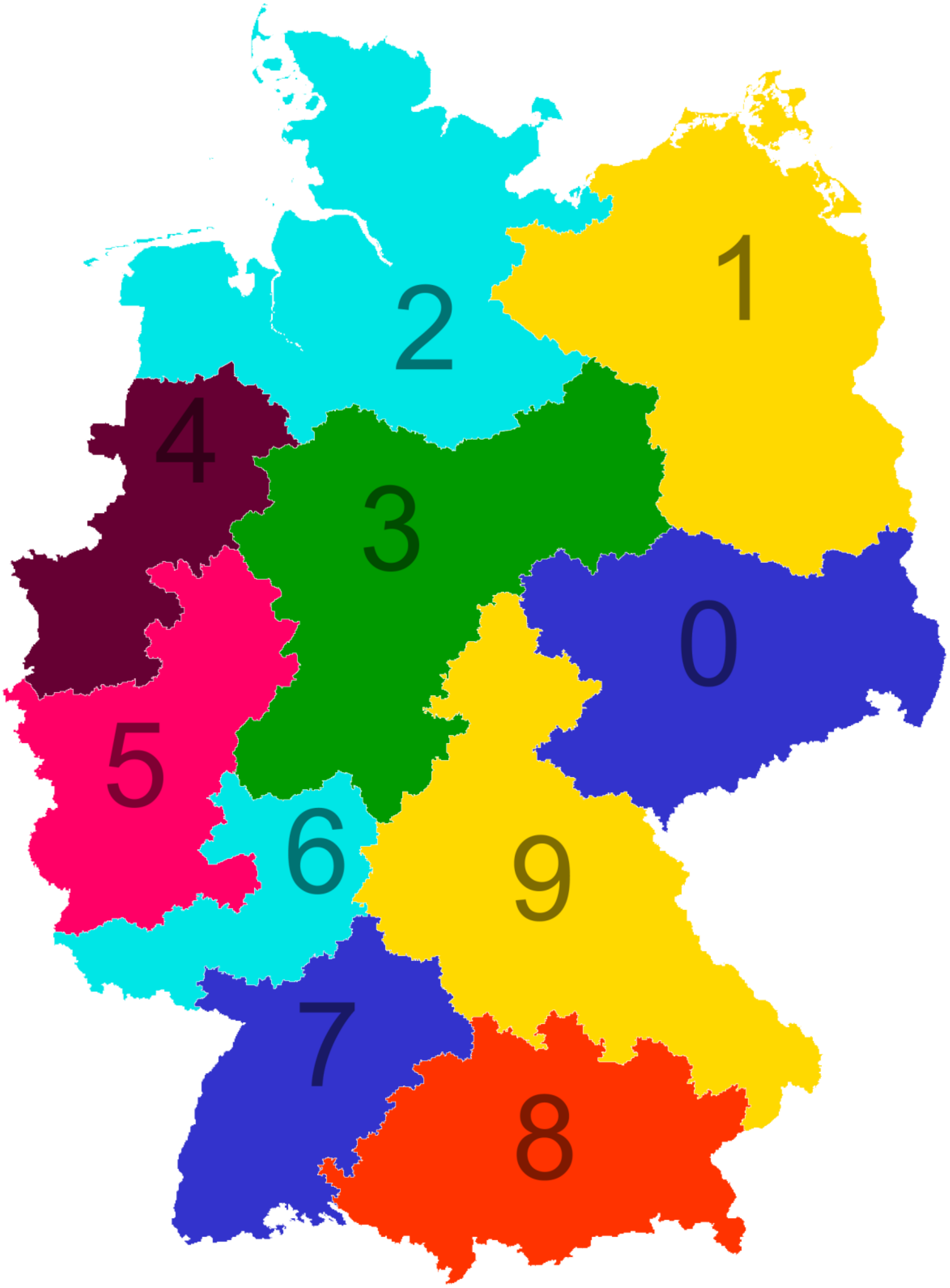

Supplement: Supplementary Material [file 23-00261_OBERLE_Supplement.pdf]
